# Urinary excretion of the acrylonitrile metabolite 2-cyanoethylmercapturic acid is correlated with a variety of biomarkers of tobacco smoke exposure and consumption

**DOI:** 10.3109/1354750X.2010.533287

**Published:** 2010-11-25

**Authors:** Emmanuel Minet, Francis Cheung, Graham Errington, Katharina Sterz, Gerhard Scherer

**Affiliations:** 1 British American Tobacco, Group Research and Development, Regents Park Road, Southampton, SO15 8TL, UK; 2 Analytisch-biologisches Forschungslabor GmbH, Goethestrasse 20, 80336 Munich, Germany

**Keywords:** Acrylonitrile, 2-cyanoethylmercapturic acid (CEMA), cigarette smoking, exposure

## Abstract

Acrylonitrile is an IARC class 2B carcinogen present in cigarette smoke. Urinary 2-cyanoethylmercapturic acid (CEMA) is an acrylonitrile metabolite and a potential biomarker for acrylonitrile exposure. The objective of this work was to study the dose response of CEMA in urine of non-smokers and smokers of different ISO tar yield cigarettes. We observed that smokers excreted >100-fold higher amounts of urinary CEMA than non-smokers. The CEMA levels in smokers were significantly correlated with ISO tar yield, daily cigarette consumption, and urinary biomarkers of smoke exposure. In conclusion, urinary CEMA is a suitable biomarker for assessing smoking-related exposure to acrylonitrile.

## Introduction

Acrylonitrile is used in the production of acrylic and modacrylic fibers, copolymers, adipinonitrile, acrylamide and other industrial chemicals (IARC, 1979; IARC, 1999). General population exposure to acrylonitrile is limited to tobacco smoke, accidental fires, and residual acrylonitrile in commercial polymeric material (Leonard *et al*., 1999). Tobacco smoke is by far the major non-occupational source for acrylonitrile exposure.

Ranges for mainstream smoke yields were reported to amount to 4.4 - 11.9 and 7.8 - 39.1 μg/cigarette when machine smoked with ISO and Massachusetts smoking parameters, respectively (IARC, 2004). The corresponding sidestream smoke yields amount to 24.1-85.6 μg/ cigarette (IARC, 2004). The ambient air concentration of acrylonitrile due to environmental tobacco smoke (ETS) was estimated to be 0.1-1.9 μg/m^3^ (Miller *et al*., 1998; Jenkins *et al*., 2000).

The International Agency for Research on Cancer (IARC) has classified acrylonitrile as a ‘possible human carcinogen’ (2B) (IARC, 1999). The evidence for this classification is mainly based on rat inhalation studies which reported nervous system, mammary, and hepatic tumors (IARC, 1999). Acrylonitrile was also shown to be mutagenic in some *in vitro* test systems, including the Ames assay (Leonard *et al*., 1999). At high doses, acrylonitrile is toxic to the central nervous system, gastrointestinal tract, and adrenals (Leonard *et al*., 1999; Thier *et al*., 2000).

**Figure 1:**
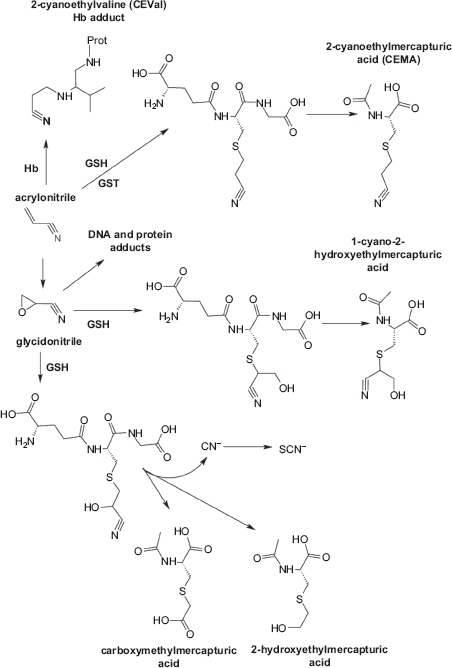
Simplified metabolic pathway of acrylonitrile, modified from Leonard *et al*., 1999. GSH:glutathione, GST: glutathione S-transferase, Hb: haemoglobin.

Once absorbed in the body, acrylonitrile is metabolized through: (i) Epoxidation to glycidonitrile, which in turn can form DNA and protein adducts and several other metabolites including cyanide (Figure 1) (Lambotte-Vandepaer *et al*., 1985; Fennell *et al*., 2000). (ii) Nucleophilic reaction with glutathione (GSH) and proteins (Figure 1) (Lambotte-Vandepaer *et al*., 1985; Fennell *et al*., 1991; Sumner *et al*., 1997; Thier *et al*., 2000).

As final products of the reaction with GSH, a number of mercapturic acids are formed which are excreted into the urine, the most important of which is 2-cyanoethylmercapturic acid (CEMA) (Figure 1) (Fennell *et al*., 1991; Sumner *et al*., 1997).

In order to test a biomarker's suitability, an assessment of specificity, sensitivity, reproducibility, and the qualification of a dose-response relationship is required. Recently, Schettgen *etal.* reported 120-fold higher urinary CEMA levels in smokers compared to non-smokers (Schettgen *et al*., 2009). Furthermore, a gradual increase in CEMA excretion was correlated to urinary cotinine (Schettgen *et al*., 2009). In 2010, Scherer and colleagues described a method to quantify acrylonitrile and other alkylating agents (Scherer *et al*., 2010). It was applied to a group of smokers of conventional cigarette products, a group of highly activated carbon filter cigarette smokers, and a group of quitters. Although a difference could be observed between the groups this work did not address the dose response relationship of CEMA as biomarker and the correlation with other cigarette smoke biomarkers.

Here, we investigated the dose-response relationship between smoking-related acrylonitrile exposure and CEMA excretion. The level of urinary CEMA in smokers of different ISO taryield cigarettes (1, 4, and 10mg)were compared with a variety of nicotine exposure biomarkers including Tneq (total nicotine equivalent) and nicotine MLE (mouth level exposure). Correlations were also established with two biomarkers of exposure to smoke toxicants, namely 3-HPMA (3-hydroxypropyl mercapturic acid), and NNAL, which are metabolites of acrolein and NNK(4-(methylnitrosamino)-1-(3-pyridyl)-1-butanone), respectively. The results presented in this report show that urinary CEMA is a robust biomarker of exposure.

## Materials and Methods

### 

#### Chemicals and standards

N-Acetyl-S-(2-cyanoethyl)-L-cysteine (2-cyanoetehylmercapturic acid, CEMA), and [d_3_]-N-acetyl-S-(2-cyanoethyl)-L-cysteine (CEMA-d_3_) were purchased from Toronto Research Chemicals, North York, Ontario, Canada. The supplier stated a chemical purity of 98 % or greater for all reference compounds. Results of this study were not corrected for purity.

Ammonium formate (> 99 %) was obtained from Sigma-Aldrich, Taufkirchen, Germany. Formic acid (98 - 100 %) and acetonitrile (for HPLC) were purchased from Merck, Darmstadt, Germany. Deionized water was prepared with Seradest equipment (Munich, Germany). All chemicals were of analytical grade or higher.

#### Analytical methods - Smoke chemistry

Three US king size commercial brands of 1, 4.7, and 10.5 mg/ cig ISO tar yields were used in this study. These products are referred as 1, 4, and 10 mg cigarettes, respectively. Smoke chemistry for the 1,4, and 10 mg ISO Tar products was analysed by GC-MS following a method adapted from Dong and colleagues (Dong *et al*., 2000). Briefly, conditioned cigarettes were smoked (three replicates) using a Borgwaldt RM20/CS smoking machine set to ISO (35 ml puff volume, 2 sec puff duration every 60 sec) and Massachusetts (45 ml puff, 2 sec puff duration every 30 sec, 50% vent holes blocked) standard smoking regimes (International Organization for Standardization, 2000; Massachusetts General Law, 1997). The generated smoke passed through a 44mm Cambridge Filter Pad (CFP), to remove particulates, before the remaining vapour phase was collected in a 3L Tedlar bag. Internal standard, deuterated acrylonitrile, was added before a precise volume (0.5mL) was injected into a GC/MS system (Agilent 6890) fitted with a RTX-VMS column (length 30 m, 0.32 mm i.d., 1.8um film thickness) for separation and analysis. Acrylonitrile was detected and quantified using a target ion at 52 m/z and two qualifier ions at 53 and 38 m/z.

#### Analytical methods - Urinary biomarkers

The determination of CEMA in urine was performed using a method adapted from Schettgen et al. (Schettgen *et al*., 2009), and validated according to the U.S. Food and Drug Administration guidelines for bioanalytical methods (FDA, 2001). In brief, 0.5 ml 50 mM ammonium formate buffer, pH 2.5 and 10 μl internal standard (IS) solution (100ng CEMA-d_3_ in 0.1 % formic acid) were added to 0.5 ml of urine vortexed and centrifuged (3000 ×g, 10min). Fifty (50) μl of this mixture was injected into an LC-MS/MS system, consisting of a Model 1200 HPLC device (Agilent Technologies, Waldbronn, Germany) and an atmospheric pressure ionization triple quadrupole mass spectrometer (Sciex API 5000, Applied Biosystems, Darmstadt, Germany). RAM (Restricted Access Material), chromatographic column, and mobile phases were as described in Schettgen *et al*., 2009. Gradient and valve positions are summarized in Table 1.

**Table 1:** Column switching and gradient program of the LC-MS/MS for CEMA in urine. Mobile phase: A: water, 0.1% formic acid; B: 40% A and 60% acetonitrile v/v.

Time (min)	Mobile phase A (%)	Valve position	Chromatographic step
0.0	80	A	RAM sample loading
0.5	80	B	Backflushing - RAM sample clean up
1.3	80	A	Injection
2.0	80	A	Separation
8.0	60	A	
10.0	50	A	
11.5	0	A	
17.0	0	A	Washing
21.0	80	A	
25.0	80	A	Reconditioning

Negative electrospray ionization (ESI-) was applied, and the MS/MS system was run in the multi-reaction monitoring (MRM) mode. Retention times as well as the quantifier and qualifier mass transitions of the analyte (CEMA) and the IS (CEMA-d_3_) are shown in Table 2. The mean quantifier/qualifier ratio was 0.7, acceptance criteria were +/-25%, which were met by all samples analyzed. LOD was estimated by extrapolating the signal to noise ratio S/N = 3 from a non-smoker urine sample with low CEMA background.

**Table 2:** Retention times (RT) and mass transitions for CEMA and the internal standard (CEMA-d_3_).

	RT(min)	Parent ion (m/z)	Daughter ion (m/z)
CEMA quantifier	13.85	215	162
CEMA qualifier		215	86
CEMA-d_3_ quantifier	13.8	218	165
CEMA-d_3_ qualifier		218	86

The method was calibrated by spiking non-smokers pooled urine with CEMA at concentrations of 2.0 to 1000ng/ml. The analyte/IS ratio of the unspiked urine (zero calibrator) was subtracted from each calibrator. Linear regression was applied for the calculation of the calibration function and the regression line was forced through the origin.

Mouth level exposure (MLE) to nicotine was estimated using previously described methodology (Shepperd *et al*., 2006; St Charles *et al*., 2006). Briefly, filter tips are collected and nicotine is extracted with methanol for quantification by gas chromatography which provides an estimate of human-smoked cigarette yields. Urinary total nicotine equivalents (Tneq) is calculated as the sum of urinary nicotine, cotinine, and trans-S'-hydroxycotinine following (3-glucuronidase treatment (Xu *et al*., 2004), 4-(Methylnitrosamino)-1-(3-Pyridyl)-1-Butanol (NNAL), and 3-hydroxypropylm-ercapturic acid (3-HPMA) were also determined, using previously described methodologies (Scherer *et al*., 2007; Shepperd *et al*., 2009).

#### Urine Samples

One hundred and ninety (190) 24h-urine samples stored at -25 °C were taken from a previous clinical study performed in Germany (Shepperd *et al*., 2009). Twenty four (24) hours urine samples were obtained from healthy smoking (n=140) and nonsmoking (n = 50) volunteers. The smokers belonged to three groups, smoking cigarettes with 10 mg (n = 47), 4mg (n = 45), or 1mg (n = 48) tar as nicotine-free dry particulate matter (NFDPM), determined according to the ISO standard smoking regime. Demographic analysis showed a normal distribution for the BMI (body mass index) with no differences between groups; however, more females (63%) were recruited in the 1 mg product category and more males were recruited in the non-smoker group (62%) and the 10 mg group (68%). The urine samples were initially collected in the course of 2006 during a residential visit to the clinic. Each group stayed at the clinic at different days to ensure compliance and limit environmental exposure for the non-smoker group. Biomarker analyses were performed in 2007 for MLE, Tneq, 3-HPMA, and NNAL and the data was reported in Shepperd *et al*., 2009. CEMA data was acquired in 2009 following analysis of the stored samples. Freeze thaw cycles and long-term storage stability for CEMA for two months (Scherer *et al*., 2010) and up to one year (data not shown) did not indicate any instability.

The study protocol and informed consent forms were approved by the Ethics Committee of the Ärztekammer Hamburg, Germany and the clinical study was conducted in accordance with the World Medical Association Declaration of Helsinki (World Medical Association, 2004) and International Conference on Harmonisation (ICH) Guidelines for Good Clinical Practice (GCP) (International Conference on Harmonization, 1996).

#### Statistics

All statistical analyses were carried out with MINITAB v15.1 (MINITAB Inc., Quality Plaza, 1829 Pine Hall Rd, State College, PA 16801-3008, USA). Summary statistics were computed for urinary CEMA, and other markers of smoke exposure (MLE to nicotine, Tneq, NNAL, 3-HPMA, and cigarettes per day) taken from the study by Shepperd *et al*., 2009. A correlation matrix was produced to test relationships across the different CEMA and markers of exposure. Analysis of variance was carried out with ISO tar yield as a factor followed by post ANOVA comparisons (Tukey's HSD test). Tukey's HSD test determines which means amongst a set of means differs from the rest (Altman., 1991).

## Results

### Smoke chemistry

To demonstrate that the selected products (1, 4, and 10mg ISO tar) yielded different amounts of acrylonitrile, mainstream smoke acrylonitrile levels were quantified under the ISO and Massachusetts smoking regime. The results are reported in Table 3 and showed an increase in acrylonitrile levels between the lower and the higher tar band products and according to smoking regime intensity.

**Table 3:** Mainstream acrylonitrile content in smoke of 1, 4, and 10 mg ISO tar products used in this study. Data is reported for the standard ISO and Massachusetts smoking regimes.

ISO Tar yields (mg/cig)	1	4.7	10.5
Acrylonitrile ISO regime (μg/cig)	0.72±0.07	2.89±0.17	7.56±0.37
Massachusetts Tar yields (mg/cig)	7	13.6	24
Acrylonitrile Massachusetts regime (ng/cig)	9.21±0.46	12.36±0.46	18.18±0.4

### Performance of the LC-MS/MS method for CEMA in urine

Performance data for the CEMA analytical method are summarized in Table 4. Ranges for intra- and inter-day precisions were 0.9 - 2.6 and 2.9 - 5.6 %, respectively. Accuracy of method was 92.4 % at high (300 ng/ ml) and 102.5 % at low (3.0ng/ml) levels. LOD and LOQ were at 0.06 and 0.17 ng/ml, respectively, allowing quantification of CEMA in all urine samples, including that of non-smokers.

**Table 4:** Method performance for the determination of CEMA in urine.

Precision	Intra-day(n = 10)	5.1ng/ml	2.60%
		150ng/m	2.00%
		278ng/ml	0.90%
	Inter-day (5 days)	5.1ng/ml	2.90%
		150ng/ml	5.60%
		278ng/ml	4.30%
Accuracy		3.0ng/ml(n = 4)	102.50%
		150ng/ml(n = 5)	98.60%
		300ng/ml(n = 3)	92.40%
LOD		0.06ng/ml	
LOQ		0.17ng/ml	
Linearity	(LOQ-ULOQ)	2.0-1000ng/ml	
		R2 = 0.99975	

### Urinary CEMA excretion in non-smokers and smokers

**Figure 2:**
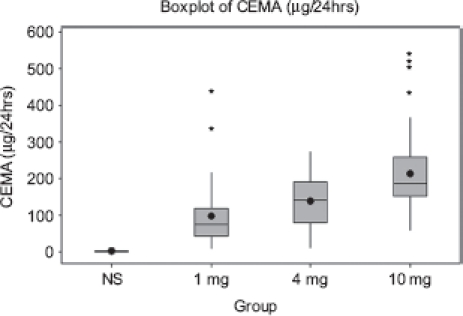
Boxplots of urinary CEMA excretion rates by smoking status and ISO tar yields of the smoked cigarettes. Circles represent means, centre lines in the boxes represent medians. The upper whisker extends to the highest data value within the upper limit (Upper limit = Q3 +1.5 (Q3 - Q1), while the lower whisker extends to the lowest value within the lower limit (Lower limit = Q1 -1.5 (Q3 - Q1)) where Q1 and Q3 are the 25th and 75th percentiles, respectively. Asterisks represent the outliers

Urinary CEMA in non-smokers and smokers of 1, 4, and 10 mg ISO tar band products was quantified using the LC-MS/MS method described above. CEMA levels in urine of non-smokers were significantly lower than in urine of smokers (Figure 2) and mean CEMA levels increased with the ISO tar levels (Figure 2) (Table 5). CEMA excretion was significantly higher in smokers of 10mg ISO tar cigarettes compared to smokers of 1mg and 4mg ISO tar cigarettes (p<0.001) (Figure 2). The difference was still significant between smokers of 1 mg and 4mg cigarettes (Figure 2). The number of cigarettes smoked daily was not significantly different between the 1 and4mggroup andthe4and 10mggroup (p>0.05),but was significantly different between the 1 and 10 mg group (Table 5). The number of cigarettes/day, however, is not the most reliable indicator of tobacco smoke exposure. Therefore the correlation between CEMA and multiple cigarette smoke exposure markers such as Tneq and nicotine MLE was also evaluated to determine a dose-response relationship.

**Table 5:** Summary statistics for CEMA and selected markers of tobacco smoke exposure in the urine of smokers and non-smokers (NS).

Variable	Group	n	Mean	StDev	Median	Tukey group
cig/day	NS	50	0	0	0	-
	1mg	48	16.8	6.6	15.0	a
	4mg	45	19.2	6.5	18.0	ab
	10mg	47	20.1	5.1	20.0	b
3-HPMA (μg/24h)	NS	50	228.4	70.4	231.6	a
	1mg	48	810.7	559.7	680.4	b
	4mg	45	1231.0	687.0	1129.0	c
	10mg	47	1945.0	968.0	1806.0	d
Total NNAL (ng/24h)	NS	50	12.7	7.5	11.1	a
	1mg	48	173.0	125.5	136.7	b
	4mg	45	274.1	144.2	276.0	c
	10mg	47	486.0	235.5	426.6	d
nicotine MLE (mg/day)	NS	50	-a	-	-	-
	1mg	48	14.0	8.7	11.5	a
	4mg	45	18.4	7.6	19.9	ab
	10mg	47	29.9	11.9	28.5	c
Tneq (mg/24h)	NS	50	0.0	0.0	0.0	a
	1mg	48	7.1	4.8	5.6	b
	4mg	45	12.5	6.2	12.1	c
	10mg	47	18.8	8.2	17.1	d
CEMA (μg/24h)	NS	50	1.3	0.7	1.1	a
	1mg	48	96.9	81.8	75.4	b
	4mg	45	139.3	72.1	140.2	c
	10mg	47	214.8	113.8	186.6	d

a MLE not relevant for non-smokers

### Correlation of urinary CEMA with other biomarkers of smoke exposure

**Figure 3:**
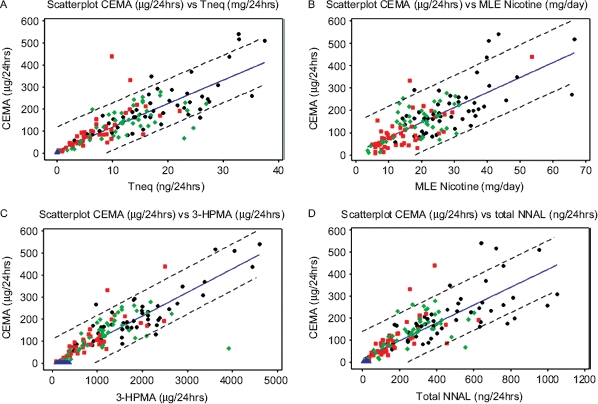
Scatterplots of CEMA versus biomarkers showing regression lines bordered by 95% prediction intervals. Key to scatterplot groups: 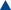 NS, 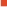
1mg, 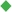
4mg, 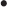 10mg. A. CEMA *vs* Tneq, B. CEMA *vs* MLE nicotine, C. CEMA *vs* 3-HPMA, D. CEMA *vs* NNAL.

Urinary CEMA levels were correlated with both, biomarkers of tobacco consumption (urinary Tneq, and nicotine MLE) and biomarkers of smoke toxicants exposure (urinary NNAL and 3-HPMA). Significant correlations (p<0.001) were found between urinary CEMA and all the biomarkers analysed. The corresponding Pearson correlation coefficients (r) are presented in Table 6 and the matrix plots for the regression are shown in Figure 3.

**Table 6:** Pearson correlation matrix between CEMA and markers of smoking dose.

Variables	cigs/day	3-HPMA (μg/24h)	Total NNAL (ng/24h)	Nicotine MLE (mg/day)	Tneq (mg/24h)
3-HPMA (μg/24h)	0.71 p < 0.001				
Total NNAL (ng/24h)	0.705 p < 0.001	0.865 p < 0.001			
Nicotine MLE (mg/day)	0.727 p < 0.001	0.806 p < 0.001	0.714 p < 0.001		
Tneq (mg/24h)	0.758 p < 0.001	0.913 p < 0.001	0.932 p < 0.001	0.761 p < 0.001	
CEMA (μg/24h)	0.738 p < 0.001	0.885 p < 0.001	0.840 p < 0.001	0.762 p < 0.001	0.869 p < 0.001

## Discussion

Biomarkers of exposure are widely regarded as the best indicators of the level of internal or absorbed dose of a toxicant in exposed subjects. Biomarkers of exposure are critical for evaluating the impact of new strategies or products that aim to reduce exposure to tobacco smoke toxicants. In this context, the development of accurate methods and careful characterization of biomarkers specificity, sensitivity, and ability to denote a dose-response relationship which is understood on a mechanistic basis is essential (IOM, 2001).

Acrylonitrile is an IARC class 2B carcinogen present in tobacco smoke which has been recommended for monitoring in tobacco products in the 2008 TobReg proposal (Burns *et al*., 2008). Jakuboswki and colleagues showed that volunteers who were experimentally exposed to 5 or 10mg/m^3^ acrylonitrile excreted, on average, 21.8 % of the retained dose as CEMA in their urine (Jakubowski *et al*., 1987). However this study was performed with only six subjects and the correlation between absorbed dose of acrylonitrile and excretion of CEMA gave equivocal results. In a recent study, Schettgen and colleagues quantified CEMA in urine of smokers (n = 81), passive smokers (n = 38), and non-smokers (n = 73) and demonstrated a good correlation between urinary CEMA and cotinine levels (Schettgen *et al*., 2009).

The aim of this work was to further evaluate CEMA as a dose-dependent biomarker of acrylonitrile exposure using 24h-urine samples of non-smokers and smokers of different ISO tar band cigarettes (1 mg, 4mg, and 10 mg). CEMA was quantified using a validated LC-MS/ MS method adapted from Schettgen *et al*, 2009.

Performance data for the method complied with the validation criteria of the US FDA (FDA, 2001). In particular, the LOQ (0.17ng/ml) was low enough to allow quantification of CEMA in background exposed non-smokers (Table 4). Furthermore, the column switching technique, which consisted of an on-line purification and concentration of the analyte prior to chromatography on a C8 analytical column, allowed full automation of the method resulting in high throughput (50–55 samples/day).

Following analysis of the urine samples, the results showed that smokers excreted between 75- and 165-fold higher amounts of the acrylonitrile biomarker CEMA than non-smokers (Table 5). Schettgen *et al*., 2009 reported urinary median CEMA levels of 2.0 μg/1 in non-smokers, 3.2-6.6 μg/1 in passive smokers, and 240 μg/1 in smokers. Absolute CEMA levels as well as smoker/non-smoker ratios (36 – 120, depending on the extent of ETS exposure) were in good agreement with the results from this study. A rough estimate of the percentage of acrylonitrile excreted as CEMA can be calculated assuming that: (i) the average smoking pattern is similar to the Massachusetts smoking regime, (ii) 50% of the acrylonitrile is retained and absorbed through the lungs (Jakubowski *et al*., 1987), (iii) the average background exposure to acrylonitrile is reflected by the CEMA level observed in non-smokers (1.3 μg CEMA/24hrs urine) and is subtracted from CEMA levels in smokers. With these assumptions, the percentage of smoking-related acrylonitrile appearing as CEMA in urine amount to 30.3, 28.5 and 28.7% which is in close agreement with the figure provided by Jakubowski *et al.* (21.8%).

Studies on the smoking-related exposure to acrylonitrile have been performed in the past with the long-term biomarker of exposure CEVal (cyanoethylvaline) haemoglobin adducts. Smokers were found to have 17- to 61-fold higher CEVal levels than non-smokers (Fennell *et al*., 1991; Bergmark, 1997; Schettgen *et al*., 2002, Scherer *et al*., 2007). This is in line with the urinary CEMA data and indicates that, with the exception of occupational exposures, acrylonitrile shows specificity for tobacco smoke exposure.

The CEMA results show a strong correlation of the acrylonitrile biomarker with various measures of smoking dose, such as daily cigarette consumption, MLE to nicotine, and urinary Tneq (r > 0.7, Table 6). This is in good agreement with the findings of Schettgen *et al*., 2009 who reported a correlation coefficient of r = 0.734 between CEMA and cotinine in urine. Although cotinine is a recognized biomarker for tobacco exposure, its level is subject to variability due to metabolic enzyme polymorphisms and ethnicity (Bramer and Kallungal, 2003). Total nicotine equivalence Tneq is a more reliable measure of nicotine consumption as it is the sum of nicotine plus five of its metabolites therefore, covering a wider range of the total nicotine mass balance. In our study a slight improvement in the correlation coefficient was observed with a value of r = 0.869 for CEMA and Tneq and r = 0.822 for CEMA and cotinine.

A significant trend in urinary CEMA levels with increasing ISO tar levels and Tneq was also observed. This appears to reflect smoke chemistry measurements, which indicates a strong association between ISO tar and ISO or Massachusetts acrylonitrile yields of the cigarettes used in this study (Table 3).

CEMA was well correlated with other biomarkers of tobacco smoke toxicants exposure such as NNAL and 3-HPMA, two metabolic products of NNK and acrolein, respectively.

The confidence intervals in Figure 3 represent the range of values inclusive of 95% of the data for all the categories confounded. For instance, in 24 hrs urine, a 20mg Tneq exposure would potentially correspond to a range of 100 to 300 μg CEMA. This illustrates the marked variability between data points, despite the strong correlation between biomarkers. A number of potential sources for the variation can be identified, including interindividual variation such as body mass index, diet, metabolism and polymorphisms, individual smoking and inhalation behavior, samples collection and storage, and instrument precision. This underlines the importance of an adequate study design to limit confounding factors.

In conclusion, the analytical method used for the determination of the urinary acrylonitrile biomarker CEMA is sufficiently sensitive and specific to detect differences between smokers and non-smokers. Urinary CEMA levels show a clear dose-response relationship to the smoking dose, such as daily cigarette consumption, MLE to nicotine and urinary Tneq. Finally, CEMA can also discriminate between smokers of different ISO tar yield cigarettes. The method is therefore appropriate to assess the quantitative changes in exposure associated with the use of tobacco products, including the switch to reduced exposure tobacco products.
